# Negative Childbirth Experience and Post-traumatic Stress Disorder - A Study Among Postpartum Women in South India

**DOI:** 10.3389/fpsyt.2021.640014

**Published:** 2021-07-06

**Authors:** Lakshmi Shiva, Geetha Desai, Veena A. Satyanarayana, Padmalatha Venkataram, Prabha S. Chandra

**Affiliations:** ^1^Department of Psychiatry, National Institute of Mental Health and Neurosciences, Bengaluru, India; ^2^Department of Clinical Psychology, National Institute of Mental Health and Neurosciences, Bengaluru, India; ^3^Department of Obstetrics and Gynecology, South Bangalore Obstetricians and Gynecologists, Rangadore Memorial Hospital, Bengaluru, India

**Keywords:** childbirth experience, traumatic childbirth, posttraumatic stress disorder, postpartum depression, India

## Abstract

**Purpose:** Negative childbirth experience has been associated with post-traumatic stress disorder (PTSD) and depression in the postpartum period with a significant impact on the mother as well as the infant.

**Methods:** The current study aimed at studying the association of negative child birth experience with PTSD and depressive symptoms among primiparous mothers within 6 weeks of child birth. The Childbirth Experience Questionnaire (CEQ), PTSD checklist (PCL), and Edinburgh Postnatal Depression scale (EPDS) were used to assess negative childbirth experience, symptoms of PTSD, and depression respectively.

**Results:** Of the 95 women in the study, fifty women (52.6%) had a score below the median of CEQ score (score of 72) indicating a negative childbirth experience. Lower Scores on CEQ indicating negative childbirth experience correlated with PTSD scores on the PCL (*r* = −0.560, *p* = 0.001) and depression scores on the EPDS (*r* = −0.536, *p* = 0.001). Of the sample of 95 women, 7.36% (*N* = 7) met the criteria for probable PTSD and 3.16% (*N* = 3) met criteria for partial PTSD. Twelve women (12.6%) had EPDS scores above 13 indicating probable clinical depression. Median CEQ scores were lower among the women who had PTSD or partial PTSD (*N* = 10) and among the women who had probable depression (*N* = 12) than those who did not. Childbirth experience emerged as the only predictor of PTSD on logistic regression (*p* = 0.03) (95% CI 1.17–79.61).

**Conclusions:** Negative childbirth experiences are common and appear to be associated with depressive and PTSD symptoms. These findings emphasize the need to provide good intrapartum care including respectful maternity care for a positive childbirth experience.

## Introduction

Birth trauma has been described as “*Any event occurring during the delivery process that involves actual or threatened serious injury or death of the mother or her infant with the birthing woman experiencing intense fear, helplessness, loss of control, and horro*r” (1, 2). It includes any event occurring during labour where the woman perceives she is stripped of her dignity (3). Terms like traumatic child birth and negative child birth experience have been the commonly used terms in the literature.

The prevalence of trauma symptoms during childbirth has been reported to be around 30–50% across studies done in varying age and income groups (4–6). Negative childbirth experience may lead to psychological problems like PTSD, depression and anxiety in the postpartum period (3, 7, 8) with PTSD symptoms being reported in nearly 20%, a diagnosis of complete PTSD in 2–6% and depressive symptoms around 20% among women after a traumatic childbirth (9–12). There is increasing evidence to suggest that PTSD after childbirth is associated with substantial functional impairment, impaired maternal fetal bonding, fear of future pregnancies and higher rates of suicidality (13, 14).

Predictors of PTSD related to childbirth include prenatal, birth related as well as post birth factors. In a meta-analysis on factors related to PTSD and child birth, prebirth vulnerability factors like antenatal depression, fear of child birth, poor health or complications in pregnancy, past history of PTSD were reported. Risk factors with birth included negative subjective birth experiences, emergency operative births, lack of support as well as dissociation during labour. Poor coping and stress was the factor related to PTSD after birth (15). History of trauma before pregnancy however had conflicting report of it being a predictor of PTSD (16, 17). Younger age (18), inadequate pain relief (10), unplanned medical interventions (19, 20), fear of delivery (21), obstetric trauma (22), postpartum depression (15), and infant health issues (23) have also been reported to be associated with PTSD. Even though prior PTSD is one of the risk factors for Postpartum PTSD, studies have shown that women without prior PTSD could also experience PTSD after a negative childbirth experience (8).

Apart from the obstetric guidelines to manage labour, the WHO lists out several recommendations on intrapartum care for a positive childbirth experience which include respectful maternity care, effective communication, companionship during labour and childbirth, and continuity of care (24). Disruptions in these domains appear to contribute to negative childbirth experience.

The current study aimed to understand the association of childbirth experience with PTSD and depression among urban Indian women who were in their postpartum period.

## Materials and Methods

### Participants

The research design was cross-sectional in nature. A purposive sample of ninety-five women who were 2–6 weeks postpartum were selected for the study from the community through an urban primary health care center and a private maternity hospital in South Bangalore, India. The inclusion criteria for the study were **-** women between the ages of 18–35 years, antenatal care or delivery at a public or private hospital in South Bangalore, primigravida (since multigravida women may be influenced by prior childbirth experience), women who have delivered a live child and in the 2–6 weeks postpartum period. Women more than 35 years of age were excluded to avoid the possible obstetric complications of being an elderly primigravida, psychological impacts of obstetric complications, or possible infertility-related psychological issues prior to conception. Women with intellectual problems, history of severe mental illness and women who have had still birth or neonatal death or serious health problems in the infant were excluded.

### Procedure

The study was conducted during the period of January to November 2016 after approval from the Institutional Ethics Committee. Women who had come for their postpartum visit or child's immunization in the state-run health centres and private hospitals located at south Bangalore were approached for the study after obtaining permission from the concerned authorities. Those who fulfilled inclusion and exclusion criteria were recruited into the study after obtaining written informed consent. Consenting women were then interviewed at the hospital outpatient department in a private room (~60%) or their homes (~40%) as per their preference.

### Measures

Sociodemographic details, namely age, education, socio-economic status, occupation, marital history along with obstetric, and infant details were collected through a structured questionnaire. Pain experience during child birth was measured on a visual analogue scale (0–10) and women were asked to rate their intensity of pain during labour.

#### Childbirth Experience Questionnaire

The CEQ is a 22-item questionnaire and includes four childbirth domains (25). These domains were derived from literature review, expert opinion of midwives and doctors as well as interviews with primiparous mothers. The domains were further named as Own capacity (8 items), Professional support (5 items), Perceived safety (6 items), and Participation (3 items). The response format for 19 questions is a 4-point Likert scale ranging from totally agree, mostly agree, mostly disagree and totally disagree, scores 1–4 are given for each item. Three other items are assessed using a visual analogue scale (VAS) and a total score is obtained from all the 22 items. Lower scores indicate a negative outcome and higher scores a positive experience. The scale was originally developed in Sweden in English language and is one among the scales that is considered valid and reliable to measure childbirth experience (26). Though, CEQ is not validated in India, it has been validated in various populations including South-Asian countries (27–32).

#### Post-traumatic Stress Disorder Checklist

PTSD was assessed using the PTSD checklist, a 17-item self-report tool, scored on a Likert scale (1–5) for assessing PTSD reflecting the DSM-4 symptoms (33–35). The minimum score on this scale would be 17 and the maximum would be 85. The cut-off score for a diagnosis of probable PTSD is 44 while a cut-off of 35 denotes probable partial PTSD. It is validated and has earlier been used in Indian women in the perinatal period (36).

#### Edinburgh Postnatal Depression Scale

Depression was assessed using the Edinburgh Postnatal Depression Scale, a 10-item self-report scale (37), each item scored from 0–3. The minimum score on this scale would be 0 and the maximum would be 30. A score of 10 or greater indicates a possible depression and 13 or greater indicates a diagnosis of depression. This scale has been validated and used widely used in several cross cultural studies, including in India (38, 39).

### Statistical Analysis

Analysis focused on the rates of negative childbirth experience, rates of full or partial PTSD and depression in this sample of women. Descriptive statistics were used for sociodemographic details, obstetric and infant details as well as scores on the PCL, EPDS, and CEQ. Spearman's correlation coefficient was used to assess the relationship between scores on the CEQ, EPDS, and PCL as the data was not normally distributed.

To understand the factors associated with PTSD, univariate analysis was done. The presence/absence of PTSD symptoms (cut-off of 35 on the PCL) was examined with various prenatal, intrapartum, and postnatal categorical covariates using Chi square test as depicted in Table 1. For the analysis of type of delivery as a factor associated with PTSD, we combined instrumental deliveries and caesarian deliveries as against vaginal delivery since the instrumental deliveries were very small in number (*N* = 2, 2.1%). Those variables which emerged as significant in the univariate analysis were used as predictors of PTSD in the forward conditional logistic regression analysis (Table 2). The statistical significance was set at *p* < 0.05 (two-tailed).

**Table 1:** Factors associated with PTSD – Univariate analysis.

**Covariates**	**Total population *N* (%)**	**PTSD symptoms absent**	**PTSD symptoms present**	**Chi-square value**	***P*-value**
**AGE, in years (mean age 24)**					
<24	54 (56.8%)	49 (51.6%)	5 (5.3%)	0.213	0.644
≥24	41 (43.2%)	36 (37. 8%)	5 (5.3%)		
**Education**					
Below graduation	52 (54.7%)	47 (49.4%)	5 (5.3%)	4.663	0.324
Graduation and above	43 (45.3%)	38 (40%)	5 (5.3%)		
**SES**					
Lower	55 (57.9%)	48 (50.5%)	7 (7.4%)	0.672	0.412
Middle/upper	40 (42.1%)	37 (38.9%)	3 (3.2%)		
**Place of delivery**					
Public hospital	42 (44.2%)	35 (36.8%)	7 (7.4%)	3.014	0.83
Private hospital	53 (55.8%)	50 (52.6%)	3 (3.2%)		
**Type of delivery**					
Vaginal	46 (48.4%)	43 (45.3%)	3 (3.2%)	4.342	0.114
Instrumental/caesarean	49 (51.6%)	42 (44.2%)	7 (7.4%)		
**Professional conducting delivery**					
Doctor	82 (86.3%)	75 (79%)	7 (7.4%)	2.519	0.112
Nurse/midwife	13 (13.7%)	10 (10.5%)	3 (3.2%)		
**Complications during delivery**					
Present	19 (20%)	16 (16.8%)	3 (3.2%)	17.612	**0.003**
Absent	76 (80%)	69 (72.6%)	7 (7.4%)		
**Pain relief**					
Received	36 (37.9%)	33 (34.7%)	3 (3.2%)	0.296	0.586
Not received	59 (62.1%)	52 (54.7%)	7 (7.4%)		
**Childbirth experience (CEQ scores)**					
Negative	50 (52.6%)	41 (43.2%)	9 (9.4%)	6.260	**0.012**
Positive	45 (47.4%)	44 (46.3%)	1 (1.1%)		
**Preterm birth**					
Present	13 (13.7%)	12 (12.6%)	1 (1.1%)	0.128	0.720
Absent	82 (86.3%)	73 (76.8%)	9 (9.4%)		
**Low birth weight**					
Present	15 (15.8%)	14 (14.7%)	1 (1.1%)	0.282	0.596
Absent	80 (84.2%)	71 (74.7%)	9 (9.4%)		
**Congenital anomalies in infant**					
Present	2 (2.1%)	2 (2.1%)	0	0.24	0.624
Absent	93 (97.9%)	83 (87.4%)	10 (10.5%)		

*The values in bold mean statistical significance*.

**Table 2:** Logistic regression – multivariate analysis.

**Variables**	**B**	**Exp B (OR)**	**95% CI**	***P*-value**
CEQ cutoff	2.268	9.659	1.172 – 79.613	**0.035**
Complications during delivery (not entered in the equation) score of 2.662	0.103			

*The values in bold mean statistical significance*.

## Results

### Sociodemographic, Obstetric, and Infant Details

The mean age of women was 24.07 years (SD = 3.86). Fifty-five women (57.9%) belonged to lower socio-economic status (SES) and the rest forty (42.1%) belonged to Middle/Upper SES. Fifty-two women (54.7%) had an education below graduation and the rest forty-three (45.3%) were graduates. All the women in the study were married. The mean number of weeks postpartum during assessment was 4.12 (SD = 1.40). The mean gestational age at delivery was 37.8 weeks (SD = 2.38). Almost half of the women delivered in public hospitals (*N* = 42, 44%) and private hospital deliveries were fifty-three (56%). Majority of women (*N* = 82, 86.4%) were delivered by the doctor and the rest (*N* = 13, 13.6%) by midwives/nurse at the hospital. Vaginal delivery occurred in 48.4% of women (*N* = 46) while instrumental delivery was 2.1% (*N* = 2) and 49.5% (*N* = 47) were caesarean deliveries.

Among the 46 women who had a vaginal delivery, 42 had undergone episiotomy. Complications during labor were noted in nineteen (20%) women which included fever, haemorrhage, readmission and prolonged labour and 15.8% of them (*N* = 15) had to be transferred to another hospital. Of the total sample, 36 women (37.9 %) received pain relief during delivery.

Mean age of the infant at the time of assessment was 4.12 weeks (SD = 1.40). Gender of the infants was equally represented with 47 (49.5%) being male and 48 (50.5%) being female. Thirteen (13.7%) infants were preterm. The mean birth weight of infants was 2.64 kgs (SD = 0.59). Low birth weight was reported in 15.8% (*N* = 15) and 2% had congenital anomalies (*N* = 2). Neonatal ICU care was received by 15.8% infants (*N* = 15).

### Details of Childbirth Experience

The median of total CEQ score was 72. The median scores on domains of CEQ were 22 (own capacity), 20 (professional support), 19 (perceived safety), and 12 (participation). When the median score of the total CEQ (72) was used as the cut-off score, almost half of the sample (52.6%, *N* = 50) had lower than the median score indicating a negative childbirth experience.

### PTSD and Depression

Of the 95 women, 7.36% (*N* = 7) met the criteria for probable PTSD (cut-off of 44 on PCL) and 3.16% (*N* = 3) met criteria for partial PTSD (cut-off of 35 on PCL). Among the women who met the criteria for PTSD symptoms (*N* = 10, 10.5%), the median CEQ was lower (37.50) compared to median CEQ of 73 in those women who did not qualify for PTSD (*N* = 85, 89.4%).

Women who scored above cut off of 13 on the EPDS (*N* = 12, 12.6%) had a lower median score of CEQ (39.5) compared to those who did not screen for depression (*N* = 83, 87.4%) with a median score of 73. Out of the 12 women (12.6%) who screened for depression, eight of them (66.6%) had more than cut-off scores on the PCL indicating postpartum depression can co-exist with PTSD following childbirth.

### Association of Sub Domain Scores of Childbirth Experience With Symptoms of PTSD and Depression

The total PCL score correlated negatively with total CEQ score (*r* = −0.560, *p* = 0.001) and also with sub-domain scores of CEQ; own capacity (*r* = −0.567, *p* = 0.001), professional support (*r* = −0.212, *p* = 0.039), perceived safety (*r* = −0.630, *p* = 0.001), and participation (*r* = −0.366, *p* = 0.001) suggesting that negative childbirth experience was associated with greater probability of PTSD symptoms.

There was also significant negative correlation between childbirth experience (CEQ scores) and depression (EPDS scores) suggesting that negative childbirth experience was associated with greater severity of depressive symptoms (*r* = −0.536, *p* = 0.001). Each of the domains of CEQ namely own capacity (*r* = −0.495, *p* = 0.001), professional support (*r* = −0.314, *p* = 0.002), perceived safety (*r* = −0.597, *p* = 0.001), and participation (*r* = −0.407, *p* = 0.001) also showed negative correlation with EPDS scores.

The pain scores captured on a VAS scale correlated negatively with CEQ scores (*r* = −0.316, *p* = 0.002) indicating that higher the intensity of pain perception, lower were the CEQ scores.

### Factors Associated With PTSD Following a Negative Childbirth Experience

On univariate analysis, various prenatal *(age, socioeconomic status, education level)*, intrapartum (*place of delivery, type of delivery, professional conducting the delivery, complications during delivery, childbirth experience and provision of pain relief*), and postnatal covariates (*infant-related factors such as preterm, low birth weight, congenital anomalies)* were analyzed for association with PTSD. None of the prenatal or postnatal factors were found to be associated with postpartum PTSD (Table 1).

Among the intrapartum factors, only ***complications during delivery***and ***childbirth*** ***experience***were found to be associated with PTSD.

Logistic regression analysis further showed that the only significant predictor of PTSD was negative childbirth experience (*p* = 0.03, 95% CI 1.17–79.61) (Table 2).

## Discussion

To the best of our knowledge, this is the first study in India which has systematically assessed the association of negative childbirth experience with PTSD and depression.

The study included a sample of women seeking antenatal care and obstetric services in public and private hospitals in South Bangalore. There was almost equal representation of women from public (44%) and private hospitals (56%) which is similar to rate of deliveries conducted in hospitals of Urban India as stated in the National Family Health Survey 4 (http://rchiips.org/nfhs/pdf/NFHS4/India.pdf). Vaginal delivery occurred in 48.4% of women (*N* = 46) while instrumental delivery was 2.1% (*N* = 2) and 49.5% (*N* = 47) were caesarean deliveries. This seems higher than the general rate of caesarian deliveries of about 28.2% reported in Urban India though rates are found to vary across public (19.9%) and private hospitals (44.8%) (40). Pain relief was received by 37.9% of women which is comparable to the acceptance rates of labour analgesia in India (41). Thirteen infants (13.7%) were preterm babies which is very similar to the preterm birth rates in India (https://www.nhp.gov.in/disease/reproductive-system/female-gynaecological-diseases-/preterm-birth). Low Birth Weight rates in India are reported to be around 25–30% (42) and our study had 15.8% (*N* = 15) low birth weight babies.

Almost half of the sample in our study (52.6%) had negative childbirth experience. Previous studies have also reported the prevalence of negative childbirth experienced by women to be in the range of 30–50% (4, 6, 9, 43). While few of the other studies have used a descriptive method or the Impact of the Trauma scale, we have used the Childbirth Experience Questionnaire (CEQ) to assess birth experience. CEQ score is a continuous variable and a study done in Iranian population used one SD less than the mean score of CEQ as the cut-off to report prevalence of traumatic childbirth of 37% (44). We have used the median of CEQ as the cut-off to state the prevalence of negative childbirth experience.

Rates of PTSD in association with negative child birth experience across other countries have been reported to vary from 1.7% (45) to 17.3% (46). The rates in our sample are similar to that reported among studies done in other Low and Middle Income countries including Nigeria (5.9%) (20) and Israel (3.4–7.9%) (10). The reasons for variation in rates of negative childbirth experience as well as PTSD in studies could be related to variations in the operational definitions of key variables, age and socioeconomic status of the participants, timing of the assessments in relation to childbirth, settings and differences in service delivery and the measures used for assessments.

Depression after childbirth was noted in 12.6% (*N* = 12) of women from our study sample, lower than previous studies (11, 12). Out of these 12 women, eight of them (66.6%) or (8.4% of the total sample) had more than cut-off scores on the PCL indicating that postpartum depression can occur as a comorbidity with PTSD following childbirth which has been reported earlier too (15).

Our results clearly showed the association of negative childbirth experience to PTSD and depression which is well-established in many other studies (3, 7, 8).

Among the co-variates assessed as predictors of PTSD (Table 1), ***complications during*** ***childbirth and negative childbirth experience***emerged as significant factors. Our study did not find age, education, SES, place of delivery, type of delivery, professional conducting delivery, pain relief or infant-related factors as significant factors for PTSD following childbirth. Though, the provision/absence of pain relief did not emerge as a predicting factor for postpartum PTSD in our study, women who had lower pain scores on the visual analogue scale reported a higher score on CEQ indicating a positive childbirth experience as reported in earlier studies (10, 47).

Complications during pregnancy and childbirth have been reported to be predictors of PTSD in various other studies (19, 20, 48) which has been also reported in our study. We had assessed for complications like fever, haemorrhage, prolonged labour, and readmission/transfer to another hospital due to the same.

Negative childbirth experience emerged as the only predictor of PTSD in our study. This finding has been reported in a previous studies (8, 15, 49). The CEQ has questions on various domains including that of professional support and perceived safety which reflect the recommendations of the WHO for intrapartum care (24). On similar lines, the Government of India had developed the LaQshya guidelines *(Labour Room Quality Improvement Initiative)* to enhance satisfaction and provide Respectful Maternity Care to all pregnant women attending health facilities. Thus, providing good intrapartum care including components of respectful maternity care, effective communication, companionship during labour and childbirth, and continuity of care can help women to perceive their childbirth as a positive experience.

The strengths of the study include ample collection of sociodemographic data, detailed assessments of negative childbirth experience in women from different backgrounds, and including women who delivered at both public and private health settings.

However, the study has a few limitations. Though, the sample included women from varying backgrounds, purposeful sampling was used which may have introduced sampling bias into the study. Prenatal factors like depression and anxiety during pregnancy and prior PTSD which may influence rates of PTSD were not studied. Only primigravida were included and social support which is often a protective factor was not examined. Detailed clinical interviews have not been done to confirm the diagnosis of PTSD or depression. Future studies using prospective cohort designs may examine these factors and how they influence the relationship between a negative childbirth experience and PTSD. Our sample size was small and therefore limits generalizability of findings. The place of interview was residence/hospital outpatient setting and could have resulted in privacy issues owing to the sensitive nature of the study.

## Conclusions

Childbirth is generally considered to be a pleasurable event in a woman's life but negative experience of childbirth often remains a tale not told. This study shows high rates of negative childbirth experience among urban women from South India who were in their postpartum period. Providing good intrapartum to women during delivery has been emphasized by many advocacies including the WHO and LaQshya programme by the Government of India which can in turn enhance positive childbirth experience. Liaison with the obstetricians, nurses, and midwives for increasing the availability of intrapartum analgesia, providing information to women and families and regularizing consent for procedures in addition to Respectful Maternity care during labour are necessary to improve maternal health care services in developing countries like India. Future studies using prospective cohort designs may examine other prenatal, antenatal as well postnatal factors to examine the association of negative childbirth experience with PTSD.

## Data Availability Statement

The datasets presented in this article are not readily available because consent from participants was taken only for this study and cannot be used for other purposes. Requests to access the datasets should be directed to dr.lakshmi0402@gmail.com or chandra@nimhans.ac.in.

## Ethics Statement

The studies involving human participants were reviewed and approved by Ethics committee of the National Institute of Mental Health and Neurosciences, Bangalore. The patients/participants provided their written informed consent to participate in this study. Written informed consent was obtained from the individual(s) for the publication of any potentially identifiable images or data included in this article.

## Author Contributions

LS the first author has contributed in data collection, analysis, and making of the draft. All the other authors have contributed in planning the research study, supervision of methodology, analysis, and preparation and proof-reading of the manuscript.

## Conflict of Interest

The authors declare that the research was conducted in the absence of any commercial or financial relationships that could be construed as a potential conflict of interest.
